# A novel three-way rearrangement involving *ETV6* (12p13) and *ABL1* (9q34) with an unknown partner on 3p25 resulting in a possible *ETV6-ABL1* fusion in a patient with acute myeloid leukemia: a case report and a review of the literature

**DOI:** 10.1186/s40364-016-0070-7

**Published:** 2016-08-25

**Authors:** Carlos A. Tirado, Ken Siangchin, David S. Shabsovich, Maryam Sharifian, Gary Schiller

**Affiliations:** 1 Department of Pathology and Laboratory Medicine, David Geffen School of Medicine at UCLA, Los Angeles, CA 90095 USA; 2 Department of Pathology and Laboratory Medicine, UCLA, West Medical Building, 1010 Veteran Ave, Second Floor room 2212F, Los Angeles, CA 90024 USA; 3 Department of Medicine, David Geffen School of Medicine at UCLA, Los Angeles, CA 90095 USA

**Keywords:** Cytogenetics, FISH, AML, MDS, *ETV6-ABL1*, Imatinib

## Abstract

**Background:**

Acute myeloid leukemia (AML) is commonly characterized by several chromosomal abnormalities resulting in the formation of chimeric genes that play various roles in leukemogenesis. Translocations resulting in the *ETV6-ABL1* fusion gene are rare in AML and other hematologic malignancies with only thirty-two previously reported cases in the literature, five of which were AML.

**Findings:**

Herein, we report the case of a 73-year-old male with acute myeloid leukemia arising from MDS, negative for *PDGFRA* and *PDGFRB*, positive for bone marrow eosinophilia, rash, and marked fluid retention, which improved dramatically with imatinib therapy. Conventional cytogenetics revealed a t(3;9)(p25;q34), t(5;18)(q13;p11.2), and additional material of unknown origin at 12p11.2 in 2 out of 10 metaphases analyzed. Interphase FISH studies showed evidence of *ETV6* (12p13) and *ABL1* (9q34) rearrangements in 41.3 % and 5.7 % of the cells respectively. FISH studies on previously G-banded metaphases showed colocalization of *ABL1* and *ETV6* signals to the short arm of chromosome 3 at 3p25 suggesting a possible *ETV6-ABL1* fusion. Subtelomeric metaphase FISH studies also showed the presence of a subtelomere 3p signal on the long arm of the derivative 9, and no subtelomere 3p signal on the derivative chromosome 12.

**Conclusions:**

These findings suggest a complex rearrangement involving an insertion of *ETV6* into 3p25 followed by a reciprocal translocation involving 3p25 and 9q34, resulting in a possible *ETV6-ABL1* fusion. This case highlights the importance of FISH to characterize complex rearrangements in myeloid malignancies, particularly those resulting in clinically significant chimeric genes.

## Introduction

The *ETV6-ABL1* fusion gene is uncommon in hematological malignancies, including chronic myeloid leukemia (CML), acute myeloid leukemia (AML), and acute lymphoblastic leukemia (ALL), and has been reported in only thirty-two patients [1]. Six reported cases of hematological malignancies bearing *ETV6-ABL1* in the context of complex rearrangements involving additional translocation partners have been reported, resulting in the translocation of the fusion gene to a third derivative chromosome [2–7]. The rarity of this event is due in part to the opposite transcriptional orientation of *ETV6* and *ABL1* relative to the centromere, which requires at least three separate chromosomal breaks to form a functional fusion gene [8]. The structure and function of the ETV6-ABL1 oncoprotein is very similar to that of the BCR-ABL1 protein with the *ETV6* helix loop helix domain (HLH) deregulating the kinase activity of *ABL1* leading to activation of a non-receptor tyrosine kinase that initiates downstream pathways affecting growth rate, cellular survival, and independence as well as transforming capacity [1, 3]. Because of the common functional activity with the BCR-ABL1 fusion protein, *ETV6-ABL1* positive patients have been observed to respond to therapy with tyrosine kinase inhibitors, albeit at varying degrees and with high likelihood of relapse [1].

Herein, we present the case of a 73-year-old male diagnosed with acute myeloid leukemia. Cytogenetic analysis revealed a t(5;18) and a t(3;9), as well as additional material of unknown origin on the short arm of chromosome 12. Additional interphase and metaphase FISH studies revealed an insertion of *ETV6* into 3p and translocation of *ABL1* to the same locus on 3p, resulting in a possible *ETV6-ABL1* fusion gene. The patient responded transiently to imatinib therapy, but eventually relapsed and expired.

## Case presentation

The patient was a 73-year-old male with acute myeloid leukemia (AML) and hypereosinophilia, arising from antecedent myelodysplastic syndrome (MDS). He was initially found to have thrombocytopenia fifteen months prior to transfer during a pre-surgical workup for surgery to treat carpal tunnel syndrome. A bone marrow biopsy performed six months later had findings consistent with myelodysplastic syndrome with fewer than 5 % blasts in the bone marrow. He subsequently received three cycles of decitabine: the first dose was given in February 2015, the second dose was given in May 2015, and the third dose was given in July 2015. Eight months after bone marrow biopsy, he presented to an outside hospital with a fever and was found to have leukocytosis with circulating blasts, and a repeat bone marrow biopsy identified AML, possibly acute eosinophilic leukemia, with 20 % blasts identified in the bone marrow. Broad-spectrum antibiotics were started and the patient was transferred to UCLA for escalation of care. Shortly after transfer, he developed progressive renal failure requiring dialysis. Persistent blasts were treated with azacytidine, but he developed severe pancytopenia. In addition, eosinophilia, rash and marked fluid retention led his clinical team to consider therapy with imatinib, which promptly led to resolution of those findings. A follow-up bone marrow aspiration and biopsy one month later identified a hypercellular marrow showing marked eosinophilia with increased atypical immature forms, markedly reduced myeloid precursors other than the eosinophilic series including increased atypical immature eosinophils, reduced erythropoiesis and megakaryopoiesis, and increased blasts (10-11 % of the marrow elements). The overall marrow histology was consistent with acute myeloid leukemia possibly, acute myelocytic leukemia.

## Material and methods

### Conventional cytogenetics

Chromosome analysis was performed using standard cytogenetic techniques on the bone marrow samples from this patient. The karyotypes were prepared using the Applied Imaging CytoVision software (Applied Imaging, Genetix, Santa Clara, CA) and described according to the ISCN 2013 nomenclature [9].

### FISH

Fluorescence *in situ* hybridization (FISH) was performed on interphase nuclei using the Vysis BCR/ABL1/ASS1 Tri-color DF FISH Probe Kit, Vysis LSI BCR/ABL Dual Color, Dual Fusion Probe Kit, and Vysis ETV6 Break Apart FISH Probe Kit from Abbott Molecular (Des Plaines, Illinois 60018) on interphase nuclei. Additionally, metaphase FISH was performed with the TotelVysion 3p, Spectrum Green, TotelVysion 3q Spectrum Orange probes, as well as the previously mentioned probes on previously G-banded metaphases.

## Results

### Conventional cytogenetics

**Fig. 1:**
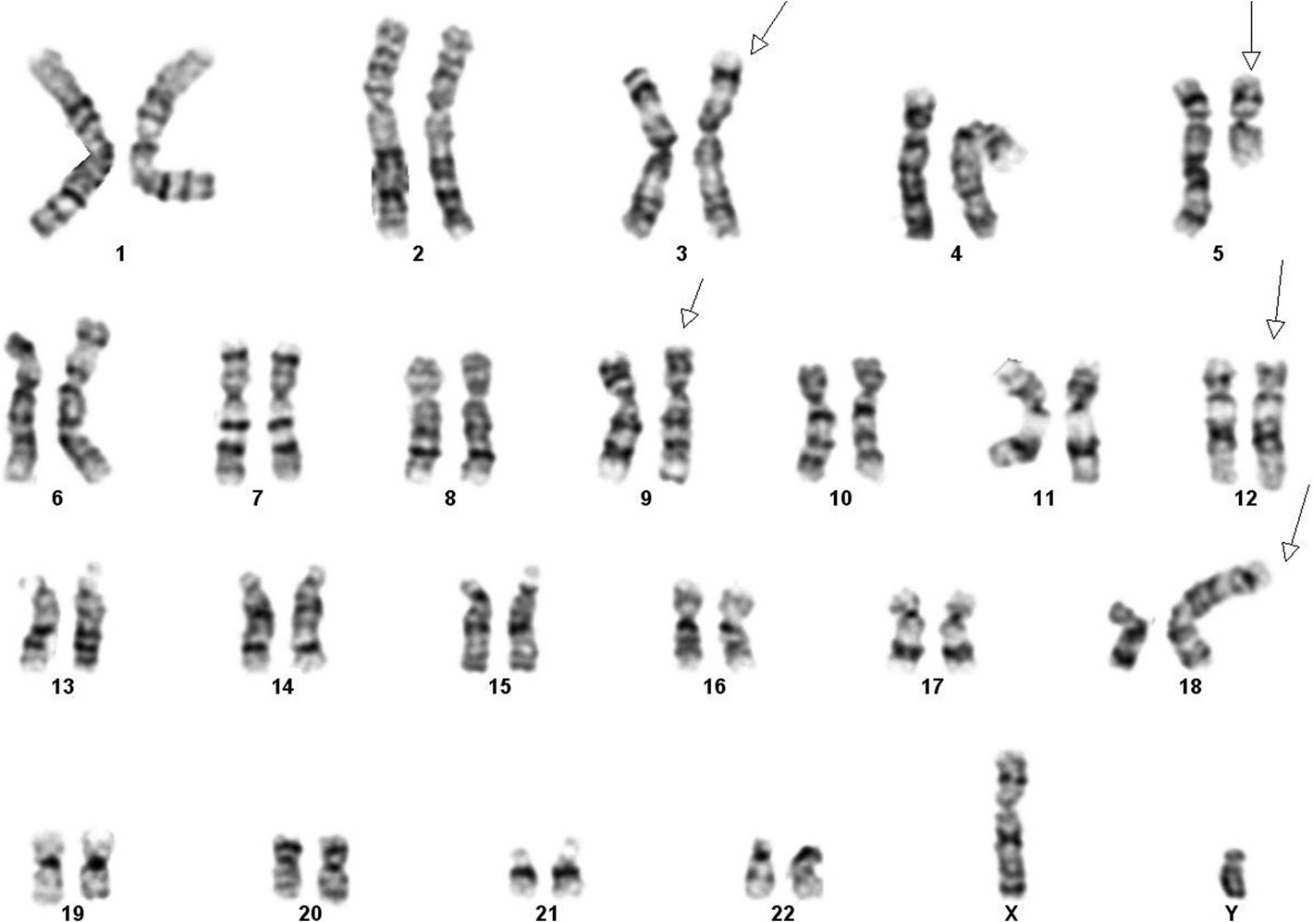
G-banded karyotype showing a three-way rearrangement involving 3, 9, and 12 as well as reciprocal translocation between chromosomes 5 and 18

Conventional cytogenetics revealed a t(3;9)(p25;q34), t(5;18)(q13;p11.2), and additional material of unknown origin at 12p11.2 in 2 out of 10 metaphases analyzed. The remaining 8 metaphases were cytogenetically normal (Fig. 1).

### FISH

**Fig. 2:**
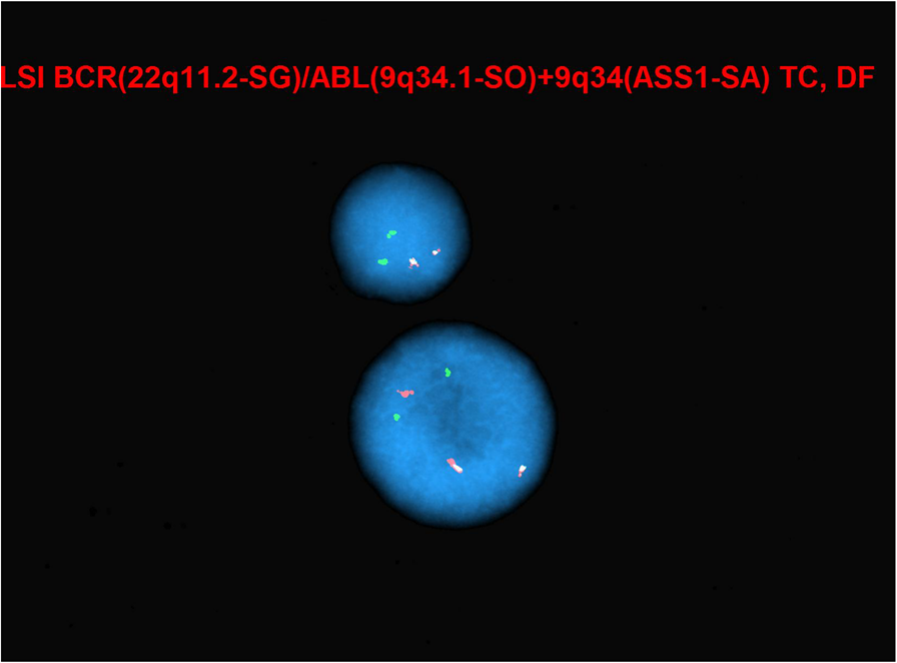
Interphase FISH showing *ABL1* rearrangement (evidenced by additional red *ABL1* signal)

**Fig. 3:**
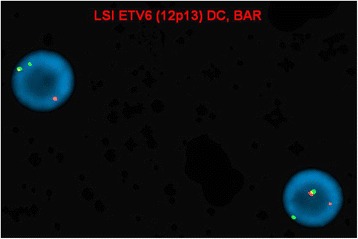
Interphase FISH showing *ETV6* rearrangement (evidenced by split red and green *ETV6* signals)

Interphase FISH studies confirmed a rearrangement in 41.3 % (124/300) of nuclei examined involving *ETV6* using the Vysis ETV6 Break Apart FISH Probe Kit and a rearrangement involving *ABL1* in 5.7 % (17/300) nuclei examined using Vysis BCR/ABL1/ASS1 Tri-color DF FISH Probe Kit. These findings were described as (Figs. 2 and 3):

- nuc ish(ASS1x2,ABL1x3,BCRx2)[17/300]
- nuc ish(ETV6x2)(5'ETV6 sep 3'ETV6x1)[124/300]

**Fig. 4:**
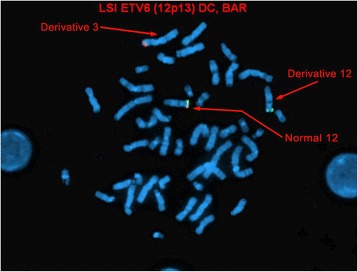
Metaphase FISH showing localization of 5’ *ETV6* red signal to the short arm of the derivative chromosome 3. The derivative chromosome 12 only shows the 3’ *ETV6* green signal

**Fig. 5:**
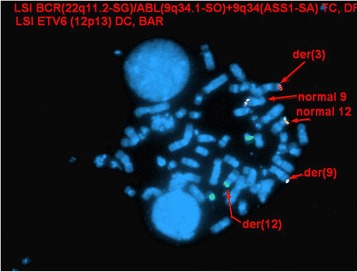
Metaphase FISH showing colocalization of *ABL1* (red signal) and 5’ *ETV6* (red signal) signals to the short arm of chromosome 3 using the BCR/ABL1/ASS1 Tri-color DF FISH Probe Kit and ETV6 Break Apart FISH Probe Kit

**Fig. 6:**
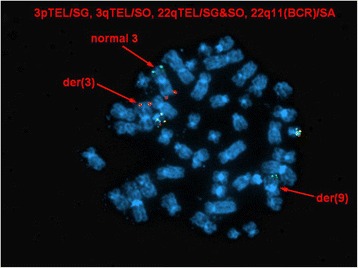
Metaphase FISH showing localization of subtelomere 3p signal on the derivative chromosome 9 and no subtelomeric 3 signals on the derivative chromosome 12

Metaphase FISH studies using the same probes on previously G-banded metaphases showed colocalization of *ABL1* and *ETV6* signals to the short arm of chromosome 3, suggesting the presence of an *ETV6-ABL1* fusion gene. Subtelomeric metaphase FISH studies also showed the presence of a subtelomere 3p signal on the derivative 9q, and no subtelomere 3p signals on the derivative 12. In light of conventional cytogenetic findings, the karyotype was conveyed as follows (Figs. 4, 5 and 6):

- 46,XY,der(3)ins(3;12)(p25;p13p13)t(3;9)(p25;q34),t(5;18)(q13;p11.2),der(9)t(3;9),der(12)ins(3;12)(p25;p13p13)add(12)(p13)[2]/46,XY[8]

## Discussion

This case highlights the formation of a potential *ETV6-ABL1* fusion gene as a result of a complex, three-way rearrangement. The conventional and molecular cytogenetic findings in this case suggest an insertion of *ETV6* in the short arm of the derivative chromosome 3 followed by a reciprocal translocation involving the same derivative chromosome 3 and *ABL1* (9q34), resulting in the potential fusion gene. The breakpoint on chromosome 3 at which the aforementioned rearrangements occurred - 3p25 - harbors *ANKRD28* (3p25.1), which has been implicated in AML in the context of t(3;11)(p25;p15) involving *NUP98* (11p15) [10]. In other studies, 3p25 was found to be the most frequently deleted chromosomal band on 3p in AML [11]. Given this information, the involvement of 3p25 in this three-way rearrangement may result in deregulation of particular target genes relevant to leukemogenesis in this region. The translocation that occurred concomitant to the three-way rearrangement - t(5;18)(q13;p11.2) - has not been observed in AML and has not been associated with any clinical or hematopathologic features.

In total, there have been thirty-two reported cases of *ETV6-ABL1* fusion gene in numerous hematologic malignancies including eleven cases of acute lymphoblastic leukemia, five cases of acute myeloid leukemia, and sixteen cases of myeloproliferative neoplasms (including CML) (Table 1). The rarity of this chromosomal rearrangement is thought to be due in part to the opposite transcriptional orientation of two genes relative to the centromere, which requires at least three break-and-join events for an in-frame fusion transcript to be formed. The rearrangement is often not detected using conventional cytogenetic techniques because of its cryptic nature due to the similar G-banding pattern of the distal long arm of chromosome 9 and the distal short arm of chromosome 12 [12]. Additionally, it has been observed that commercially available *ABL1* FISH probes may not detect aberrations in the gene in this context, suggesting that the abnormality may remain undetected in a number of cases. In interphase cells, for example, the resulting *ABL1* signal can be disproportionately small and can potentially be considered as noise and disregarded [1]. Thus, the rarity of the *ETV6-ABL1* fusion is not only due to the multi-step mechanism required for its formation, but also because of technological limitations of FISH probes and molecular cytogenetic analysis.

**Table 1:** Reported cases of *ETV6-ABL1* fusion in acute lymphoblastic leukemia (ALL), acute myeloid leukemia (AML), and myeloproliferative neoplasm (MPN), including chronic myelogenous leukemia (CML)

Reference	Age/Sex	Karyotype	FISH	Transcript
Acute lymphoblastic leukemia (ALL)				
[23]	22 mo/F	N/A	N/A	Type A
[24]	4 yo/M	47,XXYc,del(6)(q15q23)[35]/47,XXYc[7]	Intact *ETV6* (5’ and 3’) on derivative 9	Type A and B
[25]	30 yo/M	45,XY,der(1)t(1;9)(q41;q?),der(9)t(9;13)(q?;q12.3),del(9p21.1 ~ p23),-13,der(16)t(16;22)(p13.3;q11.2)	*ABL1* signal on apparently normal chromosome 12	Type A and B
[26]	8 yo/F	N/A	N/A	Type A and B
[7]	8 yo/M	46,XY, der(1)inv(1) (p11p34.2)t(1;9)(p11;p21)del(1)(q41),der(9)t(9;12) (q34.3;p13.3),der(9)t(1;9)(p11;p21),der(12)t(1;9;12) (q41?;q34.3;p13.3)[14]/46,XY)[5]	Normal signal patterns	Type A and B
[7]	5 yo/M	46,XY[20]	Normal signal patterns	Type A and B
[7]	33 yo/F	46,XX,t(8;9;12)(p12;q34;p13)[22]	*ABL1* signal on 12p	Type A and B
[13]	25 yo/F	46,XX,del(9)(p22),der(10)t(9;10)(q22; p15)[12]/46,XX[8]	N/A	Type A and B
[27]	58 yo/M	46,XY,t(9;12)(q34;p13)[2]/45,XY, −2,−14,+17[2]/46,XY[16]	N/A	Type A and B
[27]	49 yo/F	46,XY,t(9;12)(q34;p13)[10]	N/A	Type A and B
[12]	30 yo/F	47,XX,+5[19]/46,XX[1]	Extra *ETV6* and *ABL1* signals in interphase nuclei	Type A and B
Acute myeloid leukemia (AML)				
[3]	81 yo/M	t(9;12;14)(q34;p13;q22) in the context of complex karyotype	*ETV6* signal on derivative 14 with concomitant deletion of other *ETV6* allele	Type B
[4]	29 yo/F	46,XY,t(8;12)(p21;p13)[15]	5’ *ETV6* and *ABL1* signals on derivative 8	Type A
[4]	48 yo/F	46,XY,t(9;12)(q34;p13)[18/20]/51,XY,+8,+9,t(9;12)(q34;p13),+12,+14,+17[2]	5’ *ETV6* signal on derivative 9	Type B
[14]	38 yo/M	49,XY,+11,t(9;12)(q34;p1?), +der(12)t(9;12),+19,der(22)t(1;22)(q21;q11)	*ETV6-ABL1* fusion signals on 2 homologues of chromosome 12	Type A and B
[13]	52 yo/M	46,XY[20]	*ABL1* signal on apparently normal chromosome 12	Type B
Myeloproliferative neoplasm (MPN), including chronic myeloid leukemia (CML)				
[28]	49 yo/M	N/A	N/A	Type B
[2]	32 yo/M	t(12;14)(p12;q11-13)[24]/46,XY[1]	5’ *ETV6* on apparently normal chromosome 9	Type B
[24]	59 yo/M	46,XY,del(6)(p21),?t(9;12)(q34;p12)[16]	5’ *ETV6* on derivative 9q	Type A and B
[29]	44 yo/F	46,XX,t(9;12)(q34;p13)[16]/46,XX[4]	5’ *ETV6* on derivative 9q	N/A
[30]	53 yo/M	46,XY[20]	Normal signal patterns	Type A and B
[20]	36 yo/M	45,XY,-7,t(9;12)(q34;q13)[2]/45,idem,t(12;13)(p12;q13)[10]/46,XY[13]	*ABL1* signal on derivative 12	Type A
[6]	72 yo/M	46,XY,t(12;17) (p11.2;p11.2)	*ABL1* and *ETV6* signals on derivative 17	Type B
[31]	65 yo/F	46,XX,t(5;9)(q13;q34)	3’ *ABL1* on 12p	Type B
[32]	57 yo/M	46,XY	3’ *ABL1* on 12p	N/A
[18]	24 yo/F	46,XX	*ETV6* signal on apparently normal chromosome 9	Type A and B
[33]	61 yo/F	46,XX,add(9)(q34)	*ETV6* signal on derivative chromosome 9	N/A
[21]	79 yo/M	46,XY	*ABL1* signal on apparently normal chromosome 12	N/A
[22]	36 yo/M	46,XY,t(9;12)(q34;p13)	Extra *ABL1* signal (interphase FISH)	Type B
[1]	46/F	46,XY,t(9;12)(q34;p13)[10]	Extra *ETV6* and *ABL1* signals (interphase FISH)	Type B
[5]	N/A	Complex rearrangement involving chromosomes 6, 9, and 12	Suggestive of *ETV6-ABL1* fusion	N/A
[34]	31 yo/M	46,XY,der(9)t(9;12)(q34;p13), del(12)(p13)[1]	*ETV6* signal on derivative 9	Type A and B

Age/sex of patient, karyotype, salient FISH findings, and type of *ETV6-ABL1* fusion transcript detected are provided

To date, five cases of AML bearing a possible *ETV6-ABL1* fusion have been reported (Table 1). Two of the cases were designated M1, two were M6, and one was not reported. One of the cases showed a straightforward t(9;12)(q34;p13) with concurrent FISH studies showing the 3’ *ABL1* signal on the 12p, albeit in the context of a complex karyotype [4]. Two of the cases showed rearrangements involving a third chromosome - Golub et al. reported a case of AML (M6) with a t(9;12;14)(q34;p13;q22) without additional FISH studies, and La Starza et al. reported a case of AML (M1) with a t(8;12)(p21;p13) that showed colocalization of 5’ *ETV6* and 3’ *ABL1* signals on 8p21 by FISH [3, 4]. The two remaining cases showed normal karyotypes by conventional cytogenetics, but showed 3’ *ABL1* signals on 12p [13, 14]. Among all of these cases, reported secondary abnormalities observed included gains of chromosomes 8, 9, 11, 12, 14, 17, and 19, as well as a t(1;22) [4, 14].

The *ETV6-ABL1* fusion includes a helix loop helix (HLH) domain of *ETV6* and tyrosine kinase domain of *ABL1*, and each domain is necessary for constitutive phosphorylation to occur [4]. Millon et al. found that mice transplanted with *ETV6-ABL1*-positive hematopoietic stem cells developed CML-like myeloproliferative disease, and that the *TEL* pointed homology oligomerization domain was essential to *ETV6-ABL1*-driven leukemogenesis [15]. It is well known that the *ABL1* kinase has altered catalytic specificity in human leukemia. Co-immunoprecipitation studies show that the *ETV6-ABL1* fusion protein tends to form complexes with *CrkL* in Ba/F3 cells, and this interaction phosphorylates *CrkL* and possibly *CrkII* [16]. However, it is known that the in vitro studies tend to have a wider range of substrates than the cellular forms [16]. Further analysis of *Crk* and *CrkL* adaptor proteins show that they play an essential role in integrating signals from a wide variety of sources such as apoptotic cells, extracellular matrix molecules, and growth factors, and there is mounting evidence to indicate that these proteins are associated with human diseases including susceptibility to pathogens and cancer [17].

*ETV6* along with five other genes, *BCR*, *ZMIZ*, *EML*, and *Nup21* form chimeric transcripts with *ABL1*. There must be a joining of the 3’ sequence of *ABL1* with the 5’ end of the partner genes, and most of these genes are associated with a wide spectrum hematologic malignancies. Despite this heterogeneity, there is likely a common pluripotent stem cell that gives rise to similar transduction pathways and transforming activity [1]. Due to diagnostic, prognostic, and treatment-related implications, these cases further underscore the use of FISH along with routine chromosome analysis to properly characterize rare, albeit clinically significant fusion genes.

Eosinophilia is a recurrent morphologic finding in *ETV6-ABL1*-positive myeloid malignancies [4]. Of the five known AML *ETV6-ABL1* positive cases, three out of the five were reported to have an increased abnormal eosinophil count, consistent with the findings in the present case. Another finding common to most of the patients was leukocytosis and out of those three cases, two had both leukocytosis and eosinophilia [3, 4, 13, 14]. Each patient was treated with chemotherapy including, cytosine-arabinoside, idarubicin, etoposide, mitoxantrone, and cytarabine. The two patients treated with imatinib responded transiently with resolved fluid retention, eosinophilia, and leukocytosis; however, full remission was not achieved [14]. There was only one patient who achieved full cytogenetic and hematological remission 20 months after undergoing allogeneic hematopoietic stem cell transplantation, which suggests its effectiveness in the treatment of *ETV6-ABL1*-positive AML patients with eosinophilia and leukocytosis [4].

Although there is limited information about the pathogenesis of myeloid neoplasms positive for *ETV6-ABL1*, chronic myeloid leukemia (CML) positive for *BCR-ABL1* has been well studied and the molecular mechanisms of leukemogenesis and courses of clinical management are established. Tyrosine kinase inhibitors (TKI) such as imatinib are effective agents for inhibiting the constitutively activated *BCR-ABL1* tyrosine kinase in CML [18]. Similarly, *ETV6-ABL1* is also known to constitutively activate the *ABL1* tyrosine kinase, leading to cell cycle deregulation and leukemogenesis [19]. Due to the similar molecular pathogenesis of *BCR-ABL1* and *ETV6-ABL1* driven leukemogenesis, TKIs have also been considered in the treatment of patients bearing the *ETV6-ABL1* fusion. Six out of eleven CML patients positive for *ETV6-ABL1* reported in the literature were treated with imatinib: three patients showed a transient favorable response followed by relapse, one patient showed significantly decreased levels of leukemic clones, and two patients treated with 400 mg/day during the chronic phase achieved complete remission [1, 6, 18, 20–22]. Of the three that relapsed, Gancheva et al. reported a case in which the patient was administered an additional TKI, nilotinib, and the patient was able to sustain a positive response following the relapse [1]. Perna et al. did further analysis on another patient who achieved complete remission post-treatment which showed that the *ETV6-ABL1* transcript became undetectable, the white blood count normalized, and expression of *C-MYC*, *ID1*, *BCL-XL*, and *NUP-98* had decreased significantly [22]. All in all, the molecular targets of *ETV6-ABL1* and *BCR-ABL1* have significant overlaps that warrant further investigation to elucidate the effectiveness of TKIs on *ETV6-ABL1* positive hematologic malignancies.

All in all, this is the sixth reported case of AML bearing the *ETV6-ABL1* fusion gene and provides additional insight into the pathogenesis of this subset of malignancies. It is particularly important to utilize complimentary cytogenetic methodologies - namely conventional cytogenetics and FISH - in order to elucidate cryptic abnormalities, which occur more frequently in this context, and to properly characterize karyotypic changes. Additionally, screening using RT-PCR as well as other methodologies has proven useful when cytogenetic analysis is unavailable or yields negative results and in the context of broad molecular screening to identify previously unreported cases. Finally, the consideration of tyrosine kinase inhibitors, particularly second-generation ones, in the treatment of *ETV6-ABL1*-positive hematological malignancies has shown varying responses, and further investigation of its utility and clinical efficacy is warranted.

## Abbreviations

ALL, acute lymphoblastic leukemia; AML, acute myeloid leukemia; CML, chronic myelogenous leukemia; FISH, fluorescence *in situ* hybridization; HLH, helix loop helix; MDS, myelodysplastic syndrome; MPD, myeloproliferative disorder; RT-PCR, reverse transcriptase-polymerase chain reaction; TKI, tyrosine kinase inhibitor
